# Damage characteristics and constitutive modeling of coal under real-time temperatures

**DOI:** 10.1371/journal.pone.0347468

**Published:** 2026-07-01

**Authors:** Yongjiang Yu, Ning Liu, Guoning Zhang, Jiaming Liu, Jijie Wei

**Affiliations:** 1 School of Mining Engineering, Liaoning Technical University, Fuxin, Liaoning, China; 2 School of Mining Engineering, Taiyuan University of Technology, Taiyuan, Shanxi, China; 3 School of Mining Engineering, Anhui University of Science and Technology, Huainan, Anhui, China; Sichuan University of Science and Engineering, CHINA

## Abstract

The thermal-mechanical coupling effects induced by thermal injection during coalbed methane extraction can readily lead to coal seam instability. To investigate the influence of real-time temperature on the damage characteristics of coal, uniaxial compression tests were conducted on coal specimens under real-time temperature conditions using the MTS 815 testing system equipped with high-temperature accessories. Combined with the PFC3D discrete element numerical model, a cross-scale analysis was performed to examine the mechanical degradation patterns and fracture evolution characteristics of the coal. Based on a temperature-load coupled damage variable approach, a segmented damage model under combined thermal-mechanical effects was developed. The results show that the peak strain of coal is positively correlated with temperature, whereas compressive strength and elastic modulus exhibit a negative correlation. Thermal damage degree is positively correlated with temperature, with thermal cracks being predominantly tensile. During loading, as temperature increases, the macroscopic failure mode gradually shifts from axial splitting to mixed tensile-shear failure. Microdamage observed in PFC simulations similarly evolves from a predominance of tensile cracks to a mixed pattern, accompanied by a significant increase in the strain range between the initiation point and the peak point, indicating enhanced ductility. To account for the thermal sensitivity and the influence of temperature on failure mechanism, a Gaussian decay function based on a Gaussian kernel function was constructed to reconstruct the post-peak curve of the traditional Weibull model, substantially improving the model’s descriptive capability. The proposed damage constitutive model is applicable to thermomechanically coupled uniaxial compression tests on coal and accurately captures the damage evolution process. The findings provide theoretical support for assessing coal seam stability during thermal injection mining.

## 1. Introduction

As an unconventional natural gas occurring in coal seams, the development and utilization of coalbed methane (CBM) have drawn considerable attention [1–3]. However, due to the low permeability and strong adsorption characteristics of coal reservoirs, conventional CBM extraction methods often suffer from low recovery efficiency [4,5]. In recent years, thermal injection has been proposed as a promising technique to enhance CBM production [6–8].This method operates by continuously injecting high-temperature media into coalbed methane-rich reservoirs, thereby reducing the adsorption capacity of the coal and promoting methane desorption, ultimately improving extraction efficiency [9–11]. In practical engineering applications, it is essential to consider the influence of temperature on the mechanical behavior and structural integrity of coal seams. Sustained high-temperature injection can induce thermal damage and even macroscopic degradation within the coal mass, potentially compromising the long-term stability of the coal seam [12–14]. Therefore, investigating the mechanical degradation characteristics and damage mechanisms of coal under real-time temperature conditions is critical for providing theoretical support for the practical implementation of thermal injection technology.

Numerous scholars have conducted extensive research on the physical changes in coal induced by temperature. Sun et al. (2022) employed scanning electron microscopy to investigate the development of pores and cracks in coal before and after heat treatment, and found that the pore volume of heat-treated coal samples first increased and then decreased [15]. Zhou et al. (2022) used mercury intrusion porosimetry to examine the pore structure of coal after heat treatment, and reported that the peak uniaxial compressive strength of coal is controlled by pore development [16]. Wang et al. (2023) conducted uniaxial compression and acoustic emission tests on coal samples after segmented heat treatment, showing that the mechanical properties of coal gradually deteriorated with increasing temperature, and the failure mode following segmented heating was primarily shear failure [17]. Xiao et al. (2024) tested coal seam sandstone after heat treatment and observed that rock porosity increased with rising temperature, with thermal cracks accelerating the deterioration of rock strength [18]. Kumar et al. (2025) studied heat-treated rock under varying temperature conditions, and found that the fracture mechanism shifted from tensile crack-dominated to shear crack-dominated as temperature changed [19]. However, most of these studies have focused on rock that had already cooled after heat treatment, making it difficult to accurately capture the true mechanical response of coal under real-time temperature conditions—a limitation that warrants further investigation.

Currently, numerical simulation has been widely applied in various rock mechanics studies, and research on thermal damage mechanisms using this approach is also abundant. Mudarisov et al. (2022) evaluated the contact parameters of discrete element contact models and revealed the regulatory role of particle-scale parameters on the macroscopic behavior of the model [20]. Li et al. (2024) successfully simulated the acoustic emission characteristics and energy dissipation patterns of granite under thermal influence by constructing a GBM model in PFC [21]. Gu et al. (2025) used PFC2D to investigate the evolution of mesoscopic thermal stresses under high-temperature conditions, finding that internal thermal stress instability is a key factor leading to rock strength degradation [22]. Yang et al. (2025) performed PFC numerical simulations of the Brazilian split test on heat-treated sandstone, confirming that temperature promotes crack formation and distribution, and that thermal cracks directly contribute to the reduction in macroscopic rock strength [23]. However, targeted numerical simulations of microcrack evolution in thermally affected coal under uniaxial compression remain insufficient.

Many researchers have incorporated thermal damage effects into classical theoretical frameworks and conducted in-depth studies on the constitutive relationships of various rock types under coupled loading and temperature conditions, thereby enriching the theoretical understanding of rock thermal damage mechanics. Zhu et al. (2022) developed a thermal damage constitutive model for granite based on the pore compaction stage to investigate the influence of heat treatment on the failure characteristics of deep-seated rocks [24]. Zhang et al. (2022) conducted triaxial creep tests on coal and established a triaxial creep model considering temperature effects [25]. Zhou et al. (2024) performed cyclic loading and unloading tests on coal under different temperature conditions and established an elastoplastic constitutive damage model based on the continuous damage medium theory that accounts for temperature effects [26]. To investigate the influence of temperature on the deformation and failure mechanisms of coal, Dong et al. (2025) conducted uniaxial compression and acoustic emission tests on heat-treated coal and proposed a uniaxial constitutive model considering temperature conditions [27]. However, given the progressive degradation characteristics of thermally affected coal, existing evolution equations struggle to accurately capture the entire damage process.

Therefore, in this study, uniaxial compression tests were conducted under various real-time temperature conditions using the MTS 815 testing system equipped with an MTS 656 high-temperature accessory. The experimental results were combined with the PFC3D numerical model to examine the microcrack evolution characteristics of thermally affected coal, enabling a comprehensive analysis of the degradation mechanisms and fracture evolution patterns of coal under the influence of temperature. Based on the results of this analysis and considering the effect of real-time temperature on the degradation mechanism of coal, a segmented damage constitutive model suitable for describing the thermomechanical coupling behavior of coal under uniaxial compression was established. This multi-scale approach provides a theoretical basis for assessing the stability of coal in thermal injection mining projects.

## 2. Experimental methodology

### 2.1. Specimen preparation and composition analysis

**Fig 1:**
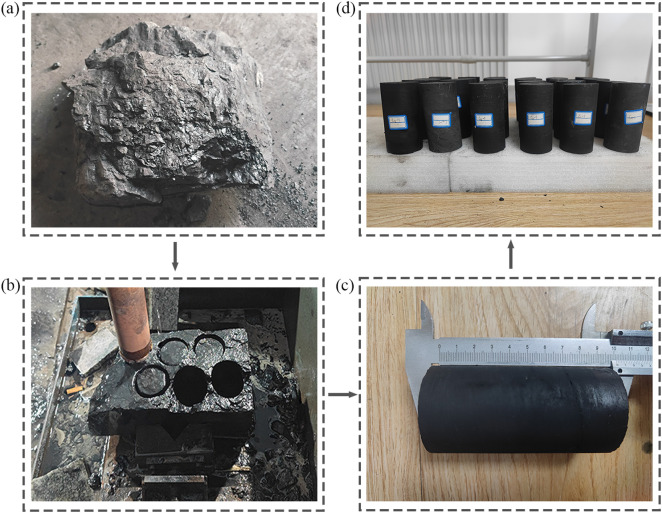
Coal sample preparation (original work by the authors).

The coal samples for this test were obtained from the Yangchangwan Coal Mine in Yinchuan City. They were prepared into standard coal-rock specimens measuring 50 × 100 mm in accordance with relevant standards. To ensure the reliability of this test, specimens without obvious cracks and with end-face parallelism deviation < 0.05 mm and height deviation between upper and lower surfaces < 0.2 mm were selected. The preparation process is shown in Fig 1.

**Fig 2:**
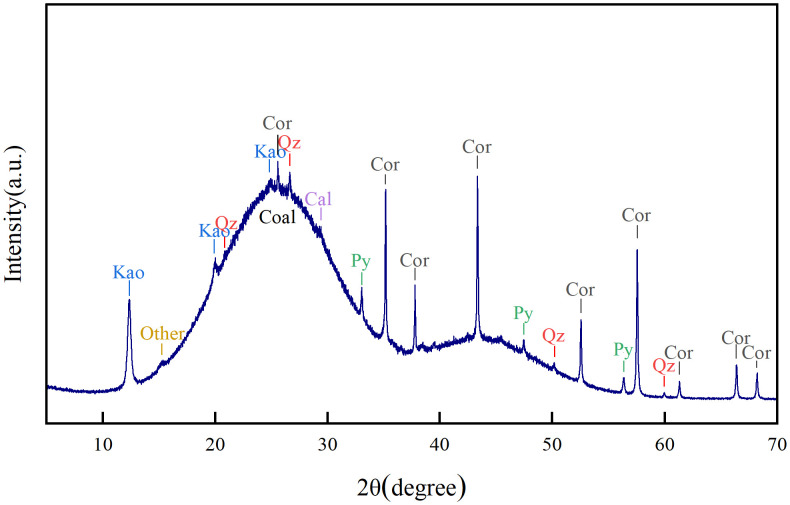
XRD pattern of the sample.


Fig 2 shows the XRD pattern of the coal sample. The relative proportions of each component in the XRD pattern were calculated using the internal standard method [28,29], with corundum serving as the internal standard at a concentration of 10%. The formula for the internal standard method is:

$$\begin{array}{c} W_{x}=\frac{I_{x}}{I_{\text{Cor}}}\times \frac{\text{1}}{RIR_{x}}\times W_{\text{Cor}} \end{array}$$ (1)

$W_{x}$ is the Mass percentage of the phase under test; *I*_*x*_ and *I*_Cor_: represent the integrals of the strongest diffraction peaks for the substance and corundum, respectively; $RIR_{x}$ is the intensity ratio of the substance relative to corundum; and *W*_Cor_ denotes the known mass fraction of the corundum standard.

XRD results indicate that the coal sample consists of the following components and their respective contents: carbon matrix (78.40%), kaolinite (12.18%), calcite (1.38%), pyrite (3.16%), and quartz (3.96%). According to the literature, no pyrolysis or phase changes occurred in these minerals within the temperature range studied in this paper [30–34].

### 2.2. Testing equipment and procedure

**Fig 3:**
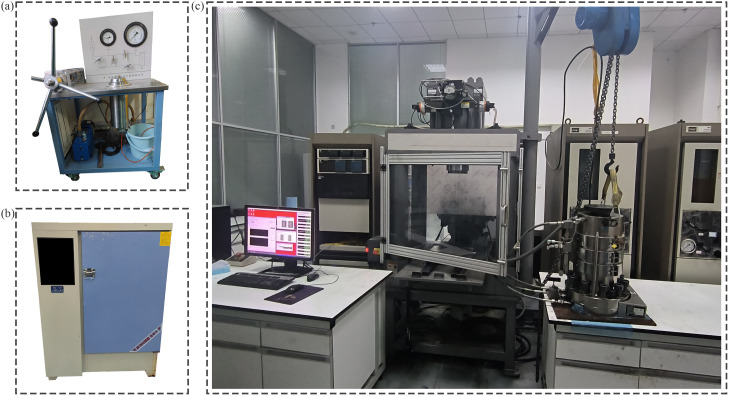
Test equipment (original work by the authors). (a) saturated water device, (b) drying apparatus, (c) MTS 815 test unit and MTS 656 accessories.

This uniaxial test was conducted using an MTS 815 testing system with displacement loading at a loading rate of 0.3 mm/min. The heating device was a Model 656 Series Triaxial Cell equipped with high-temperature components, capable of providing a stable and uniform temperature field to meet the sample’s heating and temperature-holding requirements during the test. The soaking device was a ZYB-II vacuum pressure saturation apparatus, and the drying equipment was an SHBY-40B constant-temperature curing oven. Fig 3 shows the test setup.

To ensure consistent moisture content, all coal specimens were saturated with water prior to testing and then dried in a constant-temperature curing cabinet set to 25 °C. Specimen preparation was considered complete when the change in mass measured over three consecutive measurements was less than 1%. The tests were conducted at six different ambient temperatures: 25 °C, 50 °C, 75 °C, 100 °C, 125 °C, and 150 °C. To minimize errors, the six temperatures were divided into three groups, with each group tested sequentially, resulting in a total of 18 tests (three repeats per temperature).

Testing began by placing the standard specimen into the MTS 656 high-temperature cell, using high-temperature grease on the contact surfaces to minimize end effects. The cell was then installed in the MTS 815 system. The specimen was heated to the target temperature at 2 °C/min and maintained there for 2 hours to achieve thermal equilibrium and eliminate internal stresses. It should be noted that during the heat treatment stage, the upper loading plate was not in contact with the sample. The sample remained unloaded and freely expanded throughout, ensuring that the initial damage was entirely driven by temperature. After this heat treatment, an initial preload was applied via the MTS 815 prior to the main loading phase, with all mechanical responses recorded in real time. Following the test, the specimen was unloaded, and the equipment was allowed to cool naturally to room temperature in preparation for the next run.

### 2.3. Analysis of test results

**Fig 4:**
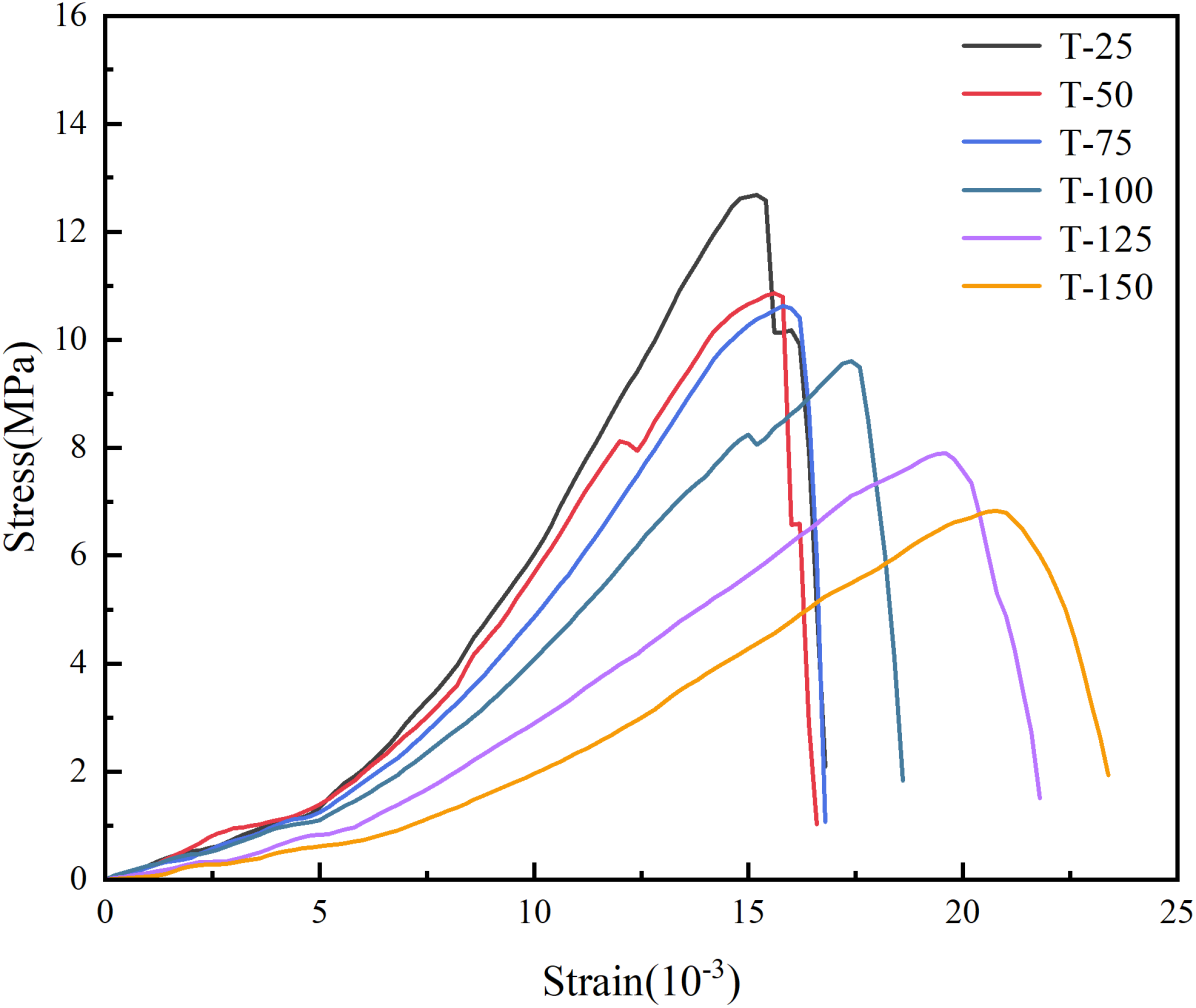
Stress-strain curves of specimens at different temperatures.

Fig 4 shows the stress-strain curves of the specimens under different temperatures; all test data are provided in S1 File. Since there is little difference between the stress-strain curves of the control group and the test group, only representative curves from the test group are included. The stress-strain curves in this paper can be divided into the following four stages: the pore and fracture consolidation stage, the linear elastic stage, the unstable fracture development stage, and the failure stage. (1) During the pore and fracture compaction stage, the coal mass’s inherent pores and voids, as well as fractures induced by temperature, cause the coal to compact under loading, resulting in an initial nonlinear concave deformation. As temperature increases, this stage gradually lengthens, indicating worsening thermal damage to the coal mass. (2) In the linear elastic stage, the stress-strain curve of the coal mass is approximately linear. As the temperature increases, the slope of this stage gradually decreases. (3) During the unstable fracture development stage, once the coal sample passes the yield point, it transitions from the elastic stage to the yield stage. Upon reaching this stage, micro-fractures in the material develop rapidly until peak strength is attained; As can be clearly observed from the curve, the peak stress gradually decreases with increasing temperature. (4) During the failure stage, after the specimen reaches its compressive strength, its internal structure is destroyed. As microcracks and cracks rapidly propagate, a macroscopic fracture surface is formed.

**Fig 5:**
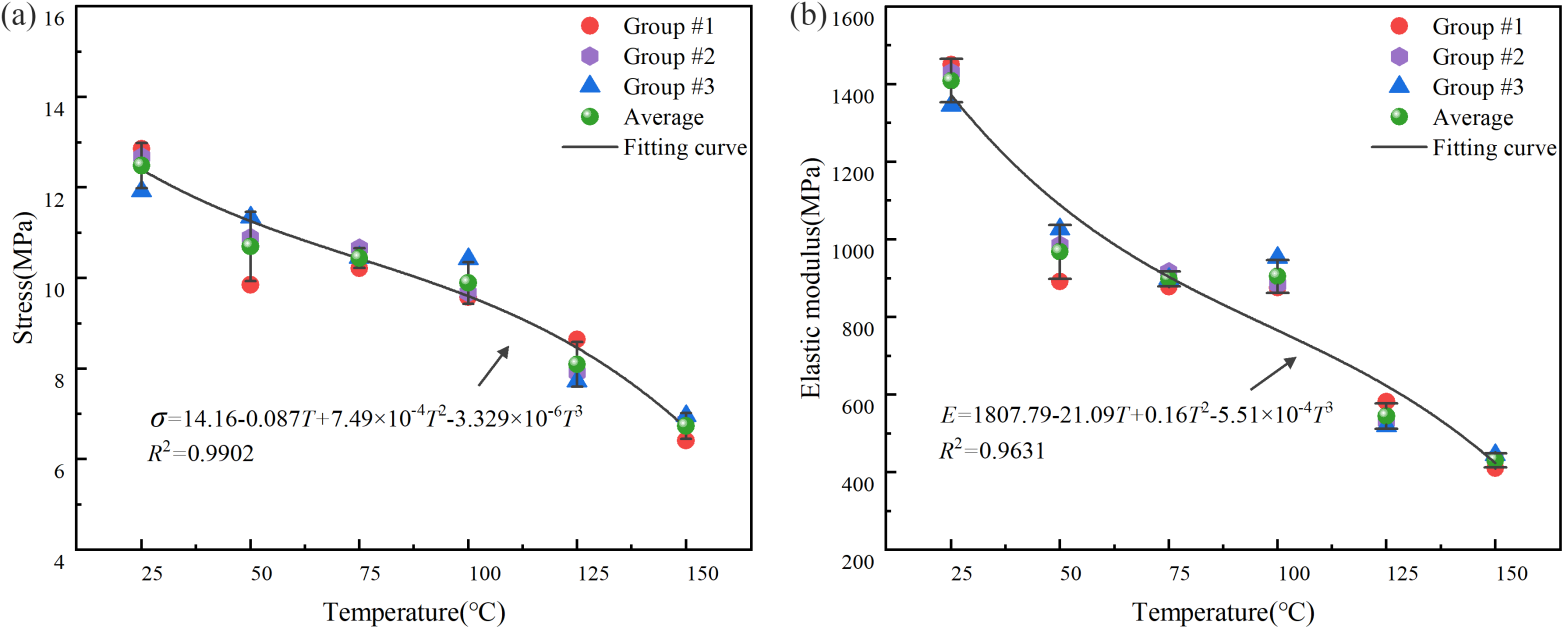
Mechanical parameters of the sample. (a) stress parameters; (b) elastic moduli parameters. Error bars represent the standard deviation of three parallel tests.

Based on the stress-strain curves of the specimens at different temperatures and considering the presence of the yield platform, the mechanical parameters were fitted using cubic functions. The results show that both the peak stress and elastic modulus of the specimens decrease with increasing temperature. To intuitively characterize the degradation of the physical and mechanical properties of coal under the coupled effects of temperature and load, this study introduces the degradation index *D*_i_. The degradation index refers to the extent to which the macroscopic mechanical properties of a material are weakened by external factors [35]. As the temperature increased from 25 °C to 150 °C, the average degradation index *D*_i_ for peak stress was 14.34%, 16.37%, 20.79%, 35.19%, and 46.10%, respectively, while that for elastic modulus was 31.32%, 36.23%, 35.81%, 61.38%, and 69.45%. These results indicate that under the influence of temperature, both the compressive strength and elastic modulus of coal decrease significantly, while the peak strain generally exhibits a positive correlation with temperature. Moreover, the deterioration of the mechanical properties of coal specimens becomes more pronounced at higher temperatures, which is consistent with the findings of Xu et al. (2022) [36] (Fig 5).

**Fig 6:**
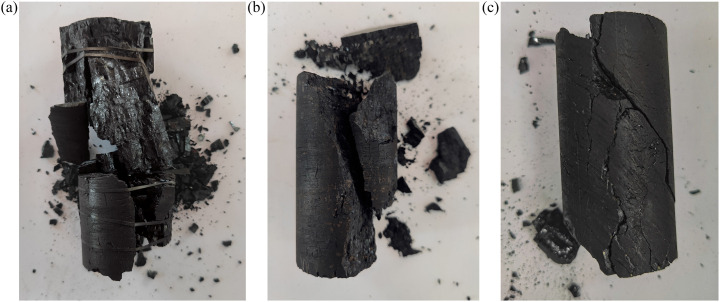
Macroscopic damage characteristics of coal specimens after testing (original work by the authors). (a) 25 °C; (b) 100 °C; (c) 150 °C.

To further elucidate the underlying physical mechanisms behind the deterioration of mechanical properties, a comparative analysis of the macroscopic fracture patterns of the specimens is presented below. It should be noted that the fracture characteristics at 50 °C and 75 °C are similar to those at 25 °C; moreover, shear bands similar to those observed at 150 °C have already begun to appear at 125 °C. To visually demonstrate the changes in fracture patterns, the fracture morphologies at three typical temperatures (25 °C, 100 °C, and 150 °C) were selected for analysis, as shown in Fig 6.

At 25 °C, multiple macroscopic cracks developed on the specimen surface, running the full length of the specimen and parallel to the axial direction. Large amounts of coal debris were scattered around the specimen, indicating that failure was accompanied by a violent energy release—a sign of highly brittle fracture. After treatment at 100 °C, the specimen broke into several large blocks rather than forming a single axial crack. Macroscopically, shear and tensile cracks coexisted, and the propagation of internal cracks became more complex. At 150 °C, the specimen exhibited clear tensile-shear composite failure, yet it remained largely intact with minimal debris spalling, suggesting a relatively moderate failure mode. The changes shown in Fig 6 indicate a shift in the failure mode of coal after heat treatment, gradually transitioning from simple tensile failure to a mixed tensile-shear pattern.

As can be seen from the above analysis, as temperature rises, thermal damage within the coal mass intensifies, thereby weakening its overall strength. This is due to the evaporation of moisture and the desorption of gases within the coal as temperature increases, both of which exert pressure on existing pores and can even induce new cracks [37]; In addition, rising temperatures cause heterogeneous thermal expansion of the minerals inside the specimen, which simultaneously creates new pores and compresses existing ones. The combined effect of these factors reduces the mechanical strength of the coal matrix [38].

## 3. PFC3D numerical simulation

Based on discrete element theory, PFC3D relies on stress-induced fracture due to microscopic bonding between particles to reveal the entire process of microcrack initiation and propagation, and can effectively handle the coupling of heat transfer and mechanical loading [39–41].Therefore, to conduct an in-depth analysis of the underlying mechanisms by which temperature affects the mechanical properties of coal rock, this study performed numerical simulations of crack evolution throughout the entire process of thermal damage and loading using PFC3D.

### 3.1. Model construction

**Fig 7:**
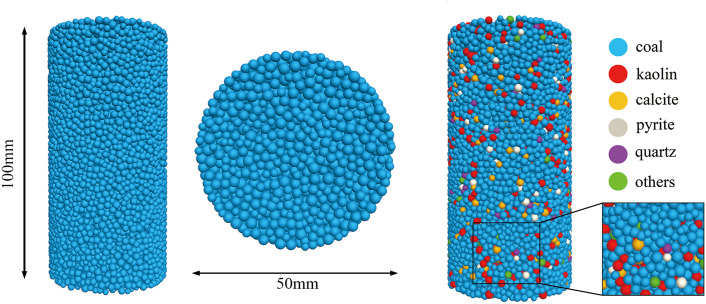
Standard coal model and mineral display.

In this study, macroscopic mechanical parameters were first used together with a trial-and-error method to calibrate the microscopic mechanical parameters of the particles. Then, within the theoretical ranges of thermal expansion coefficients from classical mineralogy handbooks and rock physics literature [42,43], the microscopic thermal expansion coefficients of the particles were iteratively calibrated in PFC. This approach accurately reproduced the macroscopic thermal-mechanical degradation characteristics of coal observed in the experiments. The specific microscopic parameters are listed in Table 1. Based on the mineral composition and these microscopic parameters, a standard coal sample model was established by assigning different physical parameters to mineral particles to reflect the differences among various minerals; all corresponding PFC simulation scripts are available in S2 File. The sample dimensions are 100 mm × 50 mm, containing a total of 18,050 particles, as shown in Fig 7.

**Table 1:** Model parameters.

Model Parameters	Numerical value	Model Parameters	Numerical value
Porosity	0.25	contact modulus/GPa	2
Minimum particle radius/mm	0.8	Parallel bond modulus/GPa	7.5
Particle size ratio	1.7	Normal strength/MPa	11
Particle density/kg/m^3^	2500	Bond shear strength/MPa	6
Friction coefficient	0.45	Bond friction angle/^o^	30
Loading rate/mm/s	0.005	Bond friction coefficient	0.3
Coal *β*	4.5 × 10^−6^	Pyrite *β*	17.0 × 10^−6^
Kaolin *β*	6.16 × 10^−6^	Quartz *β*	24.3 × 10^−6^
Calcite *β*	37.8 × 10^−6^	Others *β*	5.5 × 10^−6^

*β*: thermal expansion coefficient, (°C)^-1^.

### 3.2. Validation of numerical models

**Fig 8:**
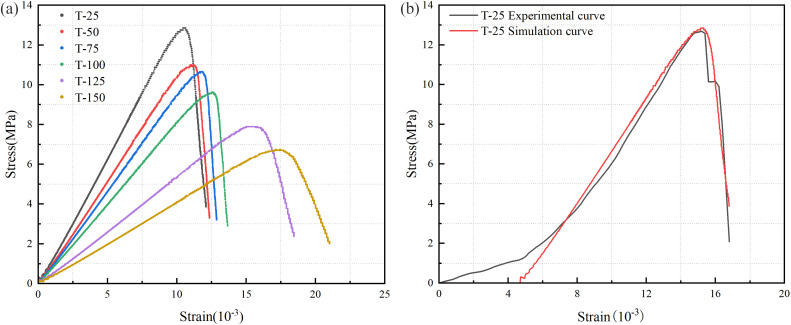
Numerical simulation stress-strain curves. (a) summary of simulated curves; (b) comparison of curves at 25 °C.

**Fig 9:**
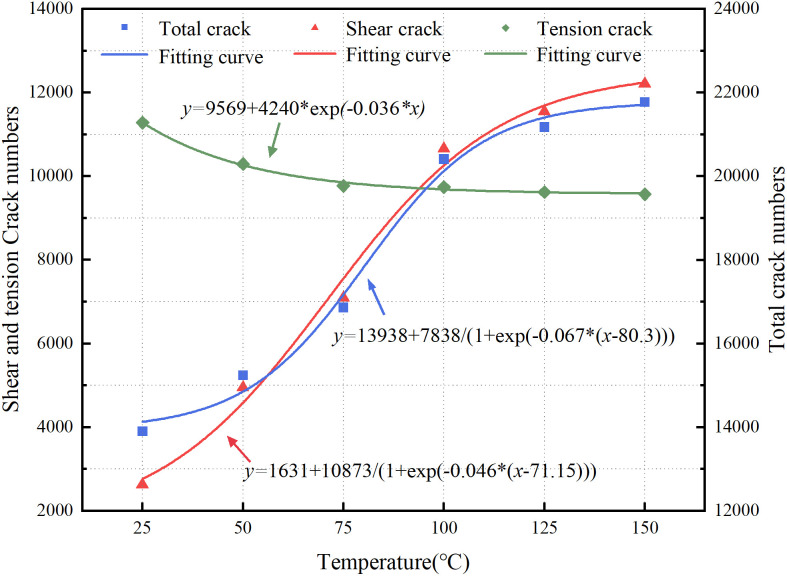
The number of coal cracks under different temperatures.

Macroscopic mechanical parameters were used for calibration, and the simulation conditions were set to match those of the experiment. A uniaxial compression simulation was then performed. The resulting simulated stress‑strain curve is presented in Fig 8, and a comparison between the measured and simulated curves is shown in Fig 9. Because the numerical simulation software PFC does not capture the consolidation stage of the coal specimen, the simulated strain values are smaller than the measured ones, and the simulated curve does not fully coincide with the experimental curve [21,44].

Table 2 lists the errors in peak strength and elastic modulus between the numerical simulation curves and the experimental stress-strain curves. The maximum error in peak stress is 0.16, the minimum error is 0.01, and the average error is 0.075; the maximum error in elastic modulus is 115.08, the minimum error is 14.76, and the average error is 61.11.

**Table 2:** Comparison of mechanical parameters between experimental tests and numerical simulations for coal specimens. Values are reported to two significant figures.

parameter	Temperature (°C)	Test value	Simulated value	error
Peak stress/MPa	25 °C	12.69	12.85	0.16
	50 °C	10.89	11.00	0.11
	75 °C	10.66	10.65	0.01
	100 °C	9.67	9.63	0.04
	125 °C	7.90	7.94	0.04
	150 °C	6.83	6.74	0.09
Elastic modulus/MPa	25 °C	1431.78	1316.70	115.08
	50 °C	986.05	1073.90	87.85
	75 °C	917.33	965.15	47.82
	100 °C	884.52	824.77	59.75
	125 °C	531.65	558.29	26.64
	150 °C	436.80	422.04	14.76

Fig 9 shows the fit of the number of cracks at the completion of uniaxial compression in the PFC simulation. Taking the second derivative of the fitting equation for the total number of cracks yields a crack growth inflection point of 80.3 °C. This point closely matches the inflection point of 79.6 °C obtained from taking the second derivative of the fitting equation for the stress parameters. These data demonstrate that the numerical simulation results are in good agreement with the experimental results, and that the use of the microstructural parameters listed in Table 1 is reasonable and feasible.

### 3.3. Analysis of thermal microcracks caused by heating

Prior to conducting the uniaxial compression test, a heat treatment simulation was performed on the model. The heating rate was set to 2 °C/min, and a heat pipe model was used to heat the particles. The outer particles began to heat up first; as time progressed, the inner particles began to heat up due to external thermal radiation and heat conduction from the surrounding particles, and all particles gradually reached the test-set temperature. When the value of the mutual interaction force between all particles fell below 1 × 10^−5^, the model was considered to have reached a state of equilibrium.

**Fig 10:**
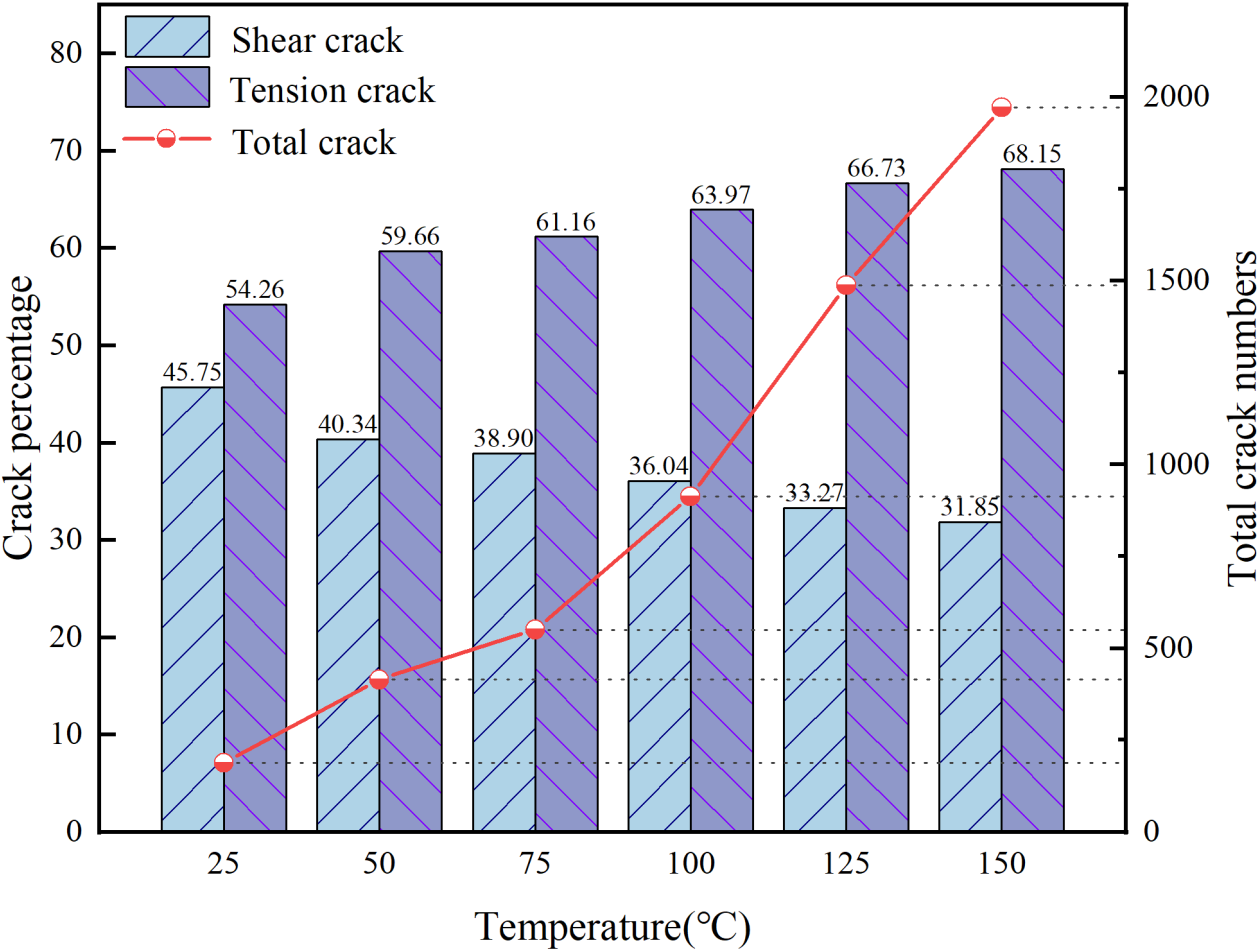
Number of microcracks after temperature treatment.

Fig 10 illustrates the change in the number of microcracks in coal under the influence of temperature. Within the temperature range studied, as temperature increases, the total number of thermal microcracks, together with the quantities of shear microcracks and tensile microcracks, all present a rising trend with an overall accelerated growth rate. This phenomenon indicates that elevated temperatures markedly aggravate the internal damage of the coal matrix and further result in the deterioration of its macroscopic mechanical properties [45]. At 25 °C, the proportions of shear cracks and tensile cracks are relatively close. As temperature rises, the proportion of shear cracks gradually decreases, and tensile cracks begin to dominate. Meanwhile, after heating, the cracks in the model are mainly concentrated in the outer regions of the specimen. These observations reflect the mechanism by which temperature influences thermal damage evolution in the material. During heating, the surface and interior of the material are heated unevenly, with the interior heating and expanding more slowly than the exterior. The exterior is constrained by internal stresses, generating tensile stress. In addition, because different minerals have different coefficients of thermal expansion, the material undergoes non-uniform thermal expansion when heated. As temperature increases, tensile stress accumulates more readily, leading to the formation of tensile cracks.

### 3.4. Microcrack evolution under uniaxial compression

**Fig 11:**
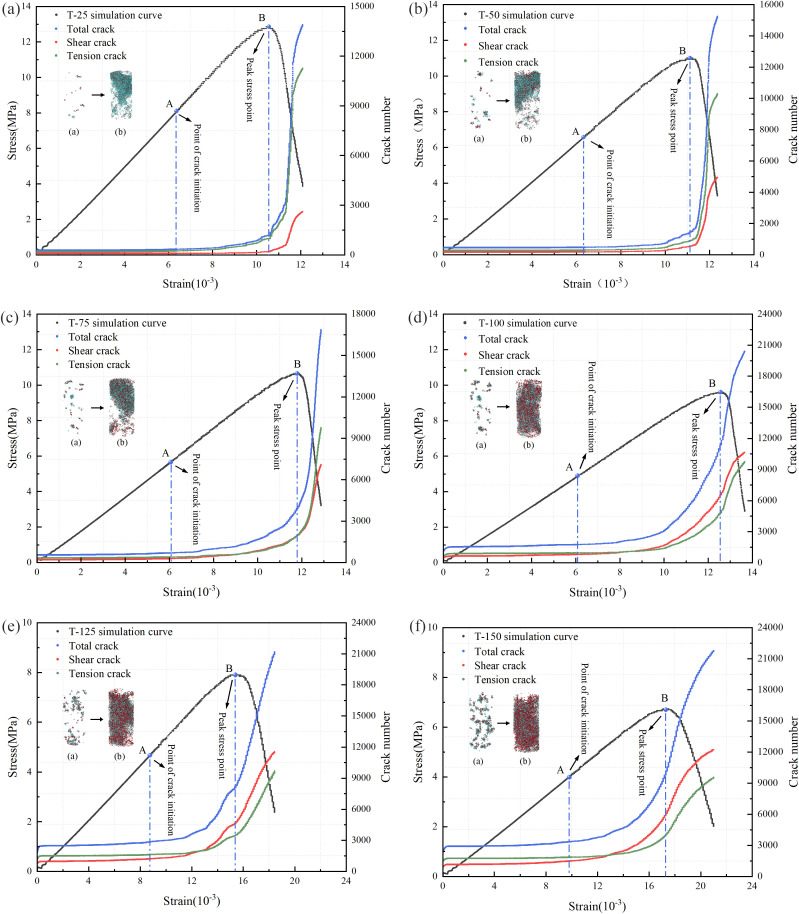
Stress-strain curves and microcrack evolution of coal at various temperatures. (a) 25 °C; (b) 50 °C; (c) 75 °C; (d) 100 °C; (e) 125 °C; (f) 150 °C.

Fig 11 shows the development trends of total cracks, shear cracks, and tensile cracks during uniaxial compression of specimens under different temperature conditions, with a microcrack image inset in the upper left corner; all relevant microcrack evolution data are provided in S1 File.

Focusing on common trends, it can be seen from Fig 11 that during the deformation and failure process of coal under different temperatures, cracks increase slightly during the initial compaction stage. Tensile cracks begin to develop near the elastoplastic transition point, and both shear and tensile cracks grow at a relatively slow rate before the stress peak, but increase significantly afterward. After the peak strength is reached, both shear and tensile cracks show a marked increase. As temperature rises, the proportion of shear cracks increases notably, eventually becoming roughly comparable to that of tensile cracks, and the failure mode gradually shifts from tensile failure to mixed shear-tensile failure.

Table 3 summarizes the changes in crack counts with temperature. As temperature rises, both the total number of cracks and the number of shear cracks in the coal matrix increase, while the number of tensile cracks decreases. The trend for shear cracks, shown in Fig 9, resembles that of total cracks, suggesting that temperature affects shear cracks in a way similar to its effect on total cracks—mainly by altering the structural stability between particles, which in turn promotes shear cracking.

**Table 3:** The number of coal cracks under different temperatures.

Types of cracks	25 °C	50 °C	75 °C	100 °C	125 °C	150 °C
Total crack	/	9.65%	10.63%	21.04%	3.74%	2.82%
Shear crack	/	88.75%	43.26%	50.42%	8.35%	5.65%
Tension crack	/	−8.73%	−5.06%	−0.31%	−1.24%	−0.58%

A closer look at the data reveals a notable shift in the damage mechanism. From 75 °C to 100 °C, shear cracks increased by 50.42%, but from 100 °C to 125 °C, the increase was only 8.35%. This sharp drop in growth rate points to a change in how the material fails. Below 100 °C, brittle behavior dominates—cracks initiate and propagate easily. Once the temperature reaches 100 °C, however, plastic deformation becomes more pronounced. Part of the energy that would otherwise generate cracks is instead consumed by irreversible deformation, making the failure process more gradual.

It should be made clear that the total cracks counted in Figs 9, 11, and Table 3 are the sum of thermal damage cracks generated during the heating stage and the cracks induced by uniaxial loading. The initial crack at zero strain in Fig 11 represents the initial damage caused by thermal effects. The increase in total cracks at high temperatures is partly due to the accumulation of thermal cracks produced during the earlier heat treatment stage, not solely from loading-induced cracks.

The change in tensile crack counts is quite distinct—they show a steady decrease as temperature rises. In Fig 11, the point where tensile cracks begin to develop shifts to the right with increasing temperature. This shift happens because thermal expansion generates compressive stress inside the material, especially near the tensile cracks caused by thermal damage, where localized compressive stress builds up. The compressive stress from thermal expansion partially counteracts the tensile stress induced by external loading, thereby raising the threshold for tensile crack initiation.

**Fig 12:**
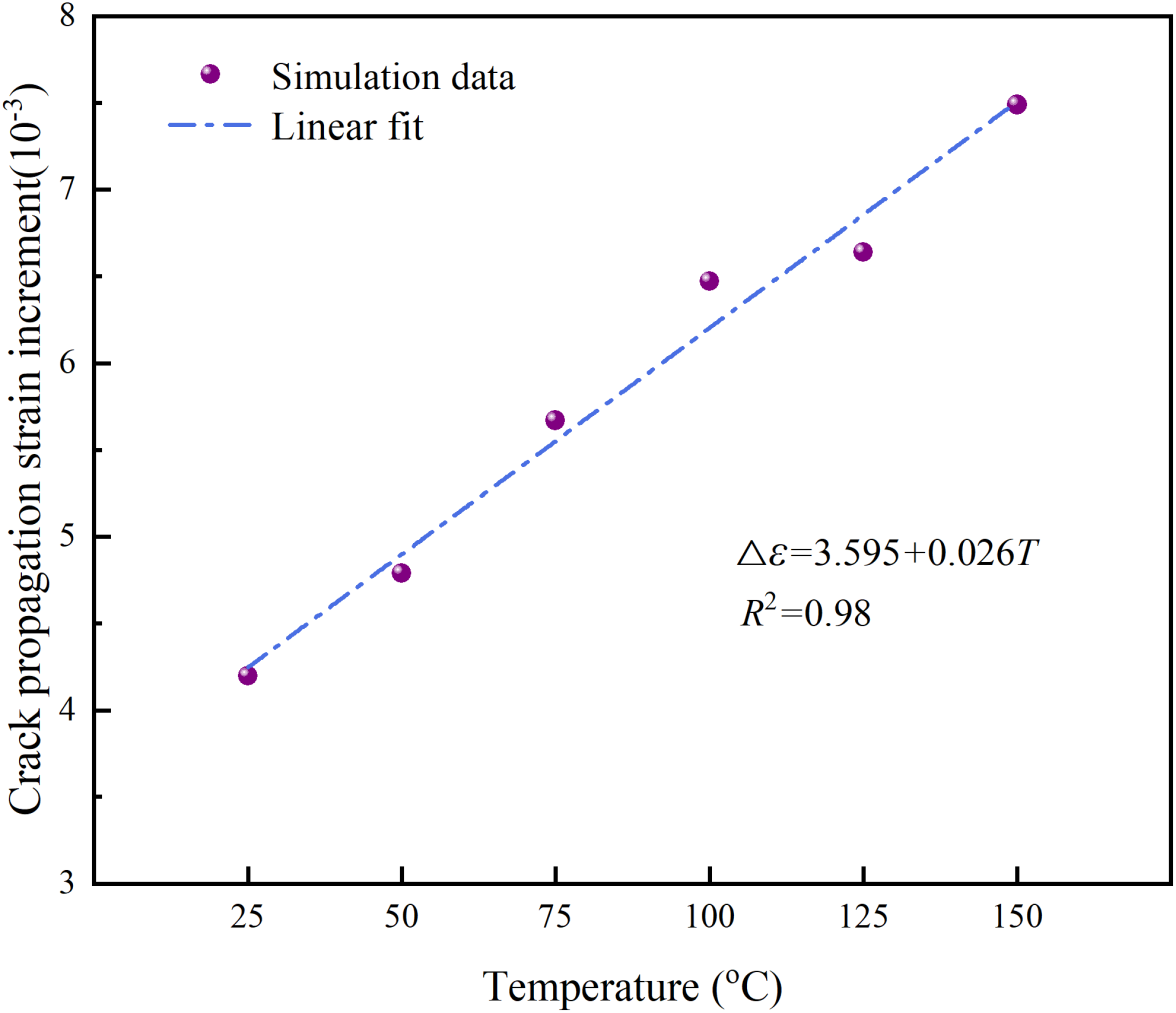
Variation in the length of the AB strain interval.

As temperature increases, the strain interval between the crack initiation point A and the peak stress point B widens. The trend in this interval is shown in Fig 12. During the pre-peak stage, the coal matrix begins to accumulate irreversible damage, and internal microcracks start to propagate rapidly. At room temperature, the coal behaves in a highly brittle manner—once cracks begin to accelerate, they quickly coalesce into a macroscopic fracture, making this stage very short in terms of strain.

After heat treatment, however, a network of randomly distributed thermal damage cracks forms within the coal. When stress-driven cracks accelerate during this stage, they frequently encounter these thermal cracks, leading to crack merging and deflection. Such complex microscopic interactions dissipate a significant amount of external energy, effectively delaying the formation of a dominant fracture surface. As a result, the coal must undergo more friction and plastic yielding before reaching its peak strength. Heat treatment therefore extends the plastic strain interval between point A and point B, promoting a transition in failure mode from brittle to ductile.

The analysis above shows that heating intensifies overall damage to the coal matrix and also alters the damage mechanism itself: the pattern shifts from being dominated by tensile cracks to a mix of tensile and shear cracking. More significantly, in the PFC simulations, the strain interval between crack initiation and peak stress widened notably with increasing temperature, showing that the coal matrix becomes increasingly ductile as temperature rises. This finding helps overcome a limitation of macroscopic stress-strain curves in capturing progressive failure, and reveals the microscopic mechanisms behind the shift in failure mode under the influence of temperature.

## 4. Thermo-mechanical damage constitutive model

The uniaxial compression tests and PFC3D simulations above show that real-time temperature significantly affects the load-bearing capacity and damage mechanisms of coal. Most traditional models assume an initial undamaged state and therefore struggle to capture the nonlinear behavior during the initial loading stage. Moreover, existing models have limitations when it comes to describing the post-peak degradation behavior of coal under thermal effects. To better characterize the damage evolution of coal under coupled temperature-loading conditions, this paper proposes a piecewise statistical damage constitutive model that accounts for nonlinear pore compaction in the initial stage and the post-peak degradation characteristics.

### 4.1. Load-induced damage

According to Lemaitre’s hypothesis of strain equivalence [46], the strain behavior of a damaged material can be described using the constitutive relations of an undamaged material—that is, effective stress can take the place of nominal stress.


$$\begin{array}{c} \sigma =\sigma^{*}(1-D)=E\varepsilon (1-D) \end{array}$$ (2)

In the equations, *σ* and σ^*^ are the nominal and effective stress tensors, respectively; *D* is the damage variable; *ε* is the strain tensor of coal; and *E* is the elastic modulus.

Based on Equation (2), Cao et al.[47] proposed a damage model that accounts for critical failure strength. Their model assumes that under loading, the load-bearing structure of coal consists of both damaged and intact parts.


$$\begin{array}{c} D=\frac{n}{N}\left(1-\frac{\sigma_{R}}{\sigma_{p}}\right) \end{array}$$ (3)

Here: *D* is the total damage variable of coal; *σ*_*R*_ is the post-failure residual strength of the specimen (MPa); *n* is the number of failed microelements; and *N* is the total number of microelements.

Given the real-time temperature loading conditions in this study, the elastic modulus in the macroscopic constitutive relation is taken as *E*(*T*), which represents the modulus of coal after degradation. The damage induced by external loading during the loading process, denoted as *D*_*p*_(*T, ε*), is defined by Equation (3). Thus, under a specific temperature condition, the constitutive relation for coal subjected only to loading is:


$$\begin{array}{c} \sigma \left(T,\varepsilon \right)=E\left(T\right)\varepsilon \left[1-D_{p}\left(T,\varepsilon \right)\right] \end{array}$$ (4)

Here: *σ*(*T, ε*) is the nominal stress under temperature conditions (MPa); *ε* is the macroscopic strain.

The Weibull distribution handles asymmetric data well and captures the stochastic nature of material failure [48], Its probability density function is expressed as follows:


$$\begin{array}{c} P\left(F\right)=\frac{m}{F_{\text{0}}}(\frac{F}{F_{\text{0}}}{)}^{m-1}exp\left[-(\frac{F}{F_{\text{0}}}{)}^{m}\right] \end{array}$$ (5)

*P*(*F*) is the distribution function of rock microelement strength; *F* is the random variable of that strength; *m* and *F*_0_ are the statistical distribution parameters of the microelements.

From the system of Equations (5), the number of failed elements, *n*, is given by:


$$\begin{array}{c} n=\int_{\text{0}}^{F}N\cdot P\left(x\right)\text{d}x=N\left\{1-exp\left[-(\frac{F}{F_{\text{0}}}{)}^{m}\right]\right\} \end{array}$$ (6)

Here, the distribution parameters m and *F*_0_ of microelement strength depend on temperature, and are thus taken as *m*(*T*) and *F*_0_(*T*). Accordingly, the load-induced damage *D*_*p*_(*T, ε*) is given by:


$$\begin{array}{c} D_{p}\left(T,\varepsilon \right)=\frac{n}{N}=1-exp\left[-{\left(\frac{F\left(T,\varepsilon \right)}{F_{\text{0}}\left(T\right)}\right)}^{m\left(T\right)}\right]\left(1-\frac{\sigma_{R}}{\sigma_{p}}\right) \end{array}$$ (7)

### 4.2. Damage under temperature utility

Previous findings show that rising temperatures cause varying degrees of thermal damage in coal, inducing crack propagation through a mechanism similar to the Arrhenius process. Macroscopically, this manifests as a nonlinear decrease in elastic modulus with increasing temperature. To capture the physical mechanism behind this temperature-induced degradation of elastic modulus, this study introduces a thermal sensitivity coefficient *α* [24], Drawing on the Arrhenius thermal activation mechanism [49–51] and dimensional normalization, an exponential evolution model for elastic modulus degradation is developed:


$$\begin{array}{c} E\left(T\right)=E_{\text{0}}\;exp\left(-\alpha \frac{T-T_{\text{0}}}{T_{max}}\right) \end{array}$$ (8)

*E*_0_ is the elastic modulus at 25 °C; *T*_0_ is 25 °C; *T*_*max*_ is the maximum temperature in this test (150 °C); and *α* is a dimensionless thermal sensitivity coefficient that reflects how sensitive the coal matrix is to temperature changes. A larger *α* means microcracks grow faster under the same temperature increase, and the mechanical properties of the coal degrade more severely.

According to continuum damage mechanics, thermal degradation of coal can be described by the damage variable *D*_*t*_(*T*), defined by the degradation of elastic modulus [52]:


$$\begin{array}{c} D_{t}\left(T\right)=1-\frac{E\left(T\right)}{E_{\text{0}}} \end{array}$$ (9)

Substituting Equation (8) into Equation (9) gives the nonlinear evolution equation for thermal damage in coal:


$$\begin{array}{c} D_{t}\left(T\right)=1-exp\left(-\alpha \frac{T-T_{\text{0}}}{T_{max}}\right) \end{array}$$ (10)

### 4.3. Temperature-load damage coupling

For coal under temperature effects, damage consists mainly of *D*_*p*_(*T, ε*) and *D*_*t*_(*T*). Thus, the total damage variable *D*_0_(*T, ε*) during coal deformation and failure is given by:


$$\begin{array}{c} D_{\text{0}}\left(T,\varepsilon \right)=D_{\text{t}}\left(T\right)+D_{p}\left(T,\varepsilon \right)\left(1-D_{\text{t}}\left(T\right)\right) \end{array}$$ (11)

Combined with the experimental and numerical results above, the damage model was revised. As shown in Fig 10, after heat treatment, a large number of thermal cracks are pre-existing inside the specimen. At the start of loading, the nonlinear closure of these thermal cracks and primary pores causes the macroscopic stress-strain curve to become concave. Therefore, a compaction coefficient *c*(*T*) (*c*(*T*) > 1) is introduced to reflect the degree of compaction of thermal cracks during the initial loading stage. The higher the temperature, the more developed the meso-thermal fractures, and the longer the macroscopic compaction stage.

With the initial modulus *E*_0_ as the reference baseline for the total damage *D*_0_(*T*, *ε*), the damage constitutive equation for coal under temperature-loading coupling is given by:


$$\begin{array}{c} \sigma \left(T,\varepsilon \right)=E_{\text{0}}\varepsilon^{c\left(T\right)}\left(1-D_{\text{0}}\left(T,\varepsilon \right)\right) \end{array}$$ (12)

By substituting formula (7), formula (8) and formula (10) into formula (12), the pre peak theoretical equation can be obtained as follows:


$$\begin{array}{c} \sigma \left(T,\varepsilon \right)=E_{\text{0}}exp\left(-\alpha \frac{T-T_{\text{0}}}{T_{max}}\right)\varepsilon^{c\left(T\right)}\left\{\frac{\sigma_{R}\text{(}T\text{)}}{\sigma_{p}}+\left(1-\frac{\sigma_{R}\text{(}T\text{)}}{\sigma_{p}}\right)exp\left[-{\left(\frac{F\left(T,\varepsilon \right)}{F_{\text{0}}\left(T\right)}\right)}^{m\left(T\right)}\right]\right\}(0\leq \varepsilon \leq \varepsilon_{p}) \end{array}$$ (13)

However, this model decays too quickly after the peak point and fails to capture the true post-peak behavior of coal. Therefore, a revised post-peak model was developed to strictly satisfy two physical conditions: (1) the stress at the peak point must be continuous and equal to the peak strength *σ*_*p*_; (2) when the strain reaches its limit at the end of the stress curve, the stress should converge to the residual strength. Under these constraints, the post-peak total stress can be decomposed into residual strength *σ*_*R*_ and a residual cohesion term that continues to decay with increasing strain $\left(\sigma_{p}-\sigma_{R}\right)$ [53–55]. In addition, the PFC simulations reveal an important trend: as temperature increases, the brittle failure characteristics of coal gradually diminish, while ductility becomes more apparent.

To mathematically capture this temperature-driven brittle-ductile transition, a Gaussian-type decay function is constructed using a Gaussian kernel function [56]:


$$\begin{array}{c} g\left(\varepsilon \right)=exp\left[-\lambda (\varepsilon -\varepsilon_{p}{)}^{\text{2}}\right] \end{array}$$ (14)

Gaussian kernel function is $f\left(x\right)=Aexp\left[-\frac{(x-\mu {)}^{\text{2}}}{2B^{\text{2}}}\right]$; In the formula, *A* is the amplitude; *B*^2^ is the variance; *μ* is the center point; *ε*_*p*_ is the peak strain; *λ* is the attenuation control parameter, satisfying *λ* = 1/2*B*^2^. The mathematical mapping shows that the smaller the λ (the larger the variance *B*^2^), the wider the curve shape of *g*(*ε*). This paper is used to describe the attenuation law of post-peak strength of materials.

The pre-peak Weibull evolution characteristics of the comprehensive formula (13) and the post-peak Gaussian type function attenuation characteristics of the formula (14) are reconstructed. The whole process segmented damage model after reconstruction is:


$$\begin{array}{c} \sigma \left(T,\varepsilon \right)=\left\{ \begin{array}{c} @l@E_{\text{0}}exp\left(-\alpha \frac{T-T_{\text{0}}}{T_{max}}\right)\varepsilon^{c\left(T\right)}\left\{\frac{\sigma_{R}\text{(}T\text{)}}{\sigma_{p}}+\left(1-\frac{\sigma_{R}\text{(}T\text{)}}{\sigma_{p}}\right)exp\left[-{\left(\frac{F\left(T,\varepsilon \right)}{F_{\text{0}}\left(T\right)}\right)}^{m\left(T\right)}\right]\right\}(0\leq \varepsilon \leq \varepsilon_{p})\\ \sigma =\sigma_{R}\left(T\right)+\left(\sigma_{p}\left(T\right)-\sigma_{R}\left(T\right)\right)exp\left[-\lambda {\left(\varepsilon -\varepsilon_{p}\left(T\right)\right)}^{\text{2}}\right]\;(\varepsilon_{p}\leq \varepsilon ) \end{array}\right. \end{array}$$ (15)

### 4.4. Solving model parameters

Due to the continuous evolution of macroscopic mechanical characteristics with temperature, the model parameters derived below are essentially state parameters with real-time temperature T as one of the independent variables, namely *α、c*(*T*), *m*(*T*), *F*_0_(*T*), *λ*.

To determine the thermal sensitivity coefficient *α*, taking the natural logarithm of both sides of Equation (8) transforms it into a linear equation:


$$\begin{array}{c} ln\sigma \left(\frac{E\left(T\right)}{E_{\text{0}}}\right)=-\alpha \frac{T-T_{\text{0}}}{T_{max}} \end{array}$$ (16)

Based on the fitted elastic modulus data in Fig 5, linear fitting was performed with $\frac{T-T_{\text{0}}}{T_{max}}$ as the abscissa and $ln\sigma \left(\frac{E\left(T\right)}{E_{\text{0}}}\right)$ as the ordinate. The absolute value of the slope of the fitted line gives the thermal sensitivity coefficient *α.*

To determine the compaction coefficient *c*(*T*), we note that thermal damage already exists before loading. Taking the heated specimen state as the new reference state for mechanical loading, the nonlinear behavior observed at the initial loading stage essentially reflects the closure of primary cracks and thermal damage cracks under load. Thus, at this stage, *D* ≈ *D*_*t*_(*T*), and the constitutive relation for nominal stress can be approximated as:


$$\begin{array}{c} \sigma \left(T,\varepsilon \right)=E_{\text{0}}\varepsilon^{c\left(T\right)}\left[1-D_{t}\left(T\right)\right] \end{array}$$ (17)

Substituting Formula (10) into Formula (17), the above formula can be simplified as:


$$\begin{array}{c} \sigma \left(T,\varepsilon \right)=E_{\text{0}}\varepsilon^{c\left(T\right)}exp\left(-\alpha \frac{T-T_{\text{0}}}{T_{max}}\right) \end{array}$$ (18)

Transform it into an outer linear equation:


$$\begin{array}{c} ln\sigma \left(T,\varepsilon \right)=c\left(T\right)ln\varepsilon +\left[lnE_{\text{0}}-\alpha \frac{T-T_{\text{0}}}{T_{max}}\right] \end{array}$$ (19)

Data points from the initial elastic compaction stage of the experimental curves at each temperature were selected, and a linear fit was performed by plotting ln*ε* against ln*σ*. The slope of the fitted line gives the compaction coefficient *c*(*T*), which incorporates thermal damage.

Based on material mechanics and Lemaitre’s equivalent strain principle, for isotropic damage induced by coal deformation, after subtracting the load-bearing part that accounts for residual strength, the relationship between nominal stress *σ*_N_ and effective stress *σ*_N_^*^ can be expressed using the total damage variable *D*_0_(*T,ε*) as:


$$\begin{array}{c} \sigma \left(T,\varepsilon \right)=\sigma^{*}\left(T,\varepsilon \right)\left[1-D_{\text{0}}\left(T,\varepsilon \right)\right] \end{array}$$ (20)

Using the pre-peak damage constitutive equation constructed above, under uniaxial compression (lateral confining stress σ^*^_2_ = σ^*^_3_ = 0), the axial effective stress *σ*_1_ can be directly expressed in terms of the macroscopic axial strain and the initial compaction coefficient as:


$$\begin{array}{c} {\sigma_{\text{1}}}^{*}\left(T,\varepsilon \right)=E_{\text{0}}\;exp\left(-\alpha \frac{T-T_{\text{0}}}{T_{max}}\right){\varepsilon_{\text{1}}}^{c\left(T\right)} \end{array}$$ (21)

Since the micro-element failure is driven by the internal real stress field, the Drucker-Prager (D-P) strength failure criterion [57,58] is used as a measure of the micro-element strength. Its expression is:


$$\begin{array}{c} F\left(T,\varepsilon \right)=\alpha_{\text{0}}{I_{\text{1}}}^{*}\left(T,\varepsilon \right)+\sqrt{{J_{\text{2}}}^{*}\left(T,\varepsilon \right)} \end{array}$$ (22)

In the formula: $\alpha_{\text{0}}=\frac{sin\varphi }{\sqrt{9+3{sin}^{\text{2}}\varphi }}$, *φ* is the internal friction angle of rock, °; *I*_1_^*^ and *J*_2_^*^ are the first invariant of the effective stress tensor and the second invariant of the effective stress, respectively. The expressions of *I*_1_^*^ and *J*_2_^*^ are:


$$\begin{array}{c} {I_{\text{1}}}^{*}={\sigma_{\text{1}}}^{*}+{\sigma_{\text{2}}}^{*}+{\sigma_{\text{3}}}^{*} \end{array}$$ (23)

$$\begin{array}{c} {J_{\text{2}}}^{*}=\frac{({\sigma_{\text{1}}}^{*}-{\sigma_{\text{2}}}^{*}{)}^{\text{2}}+({\sigma_{\text{2}}}^{*}-{\sigma_{\text{3}}}^{*}{)}^{\text{2}}+({\sigma_{\text{3}}}^{*}-{\sigma_{\text{1}}}^{*}{)}^{\text{2}}}{\text{6}} \end{array}$$ (24)

Here: *σ*_1_^*^, *σ*_2_^*^, *σ*_3_^*^ is the effective stress, MPa.

Substituting the effective stress boundary conditions under uniaxial compression into Equations (23) and (24) gives the effective stress invariants as:


$$\begin{array}{c} {I_{\text{1}}}^{*}\left(T,\varepsilon \right)={\sigma_{\text{1}}}^{*}\left(T,\varepsilon \right) \end{array}$$ (25)

$$\begin{array}{c} \sqrt{{J_{\text{2}}}^{*}\left(T,\varepsilon \right)}=\frac{\text{1}}{\sqrt{\text{3}}}{\sigma_{\text{1}}}^{*}\left(T,\varepsilon \right) \end{array}$$ (26)

Substituting Equations (25) and (26) into the D‑P criterion under effective stress (Equation (22)) yields the expression for the microelement strength tensor under uniaxial compression:


$$\begin{array}{c} F\left(T,\varepsilon \right)=\left(\alpha_{\text{0}}+\frac{\text{1}}{\sqrt{\text{3}}}\right){\sigma_{\text{1}}}^{*}\left(T,\varepsilon \right) \end{array}$$ (27)

Substituting the effective stress-strain relationship from Equation (21) into Equation (27) yields the explicit evolution equation for the microelement strength of coal as a function of macroscopic strain:


$$\begin{array}{c} F\left(T,\varepsilon \right)=\left(\alpha_{\text{0}}+\frac{\text{1}}{\sqrt{\text{3}}}\right)E_{\text{0}}exp\left(-\alpha \frac{T-T_{\text{0}}}{T_{max}}\right)\varepsilon^{c\left(T\right)} \end{array}$$ (28)

For the uniaxial test stress-strain curve, the coal body is destroyed at the peak point, and the condition is satisfied at the peak point (*ε*_*p*_*,σ*_*p*_), and the derivative is strictly 0, so it satisfies the extreme continuous condition:


$$\begin{array}{c} \frac{\partial \sigma \left(T,\varepsilon \right)}{\partial \varepsilon }|\sigma =\sigma_{p}\left(T\right),\varepsilon =\varepsilon_{p}\left(T\right)=0 \end{array}$$ (29)

In order to simplify the expression, the theoretical elastic bearing stress ratio at the peak point is *W*(*T*)*:*


$$\begin{array}{c} W\left(T\right)=\frac{\sigma_{p}}{E_{\text{0}}\;exp\left(-\alpha \frac{T-T_{\text{0}}}{T_{max}}\right){\varepsilon_{p}}^{c\left(T\right)}} \end{array}$$ (30)

Substituting Equation (13) into Equation (29) and solving yields the parameters *m*(*T*) and *F*_0_(*T*) as:


$$\begin{array}{c} m\left(T\right)=\frac{W\left(T\right)}{\left(W\left(T\right)-\frac{\sigma_{R}\left(T\right)}{\sigma_{p}}\right)\left\{ln\left[1-\frac{\sigma_{R}\left(T\right)}{\sigma_{p}}\right]-ln\left[W\left(T\right)-\frac{\sigma_{R}\left(T\right)}{\sigma_{p}}\right]\right\}} \end{array}$$ (31)

$$\begin{array}{c} F_{\text{0}}\left(T\right)=\frac{\left(\alpha_{\text{0}}+\frac{\text{1}}{\sqrt{\text{3}}}\right)E_{\text{0}}\;exp\left(-\alpha \frac{T-T_{\text{0}}}{T_{max}}\right){\varepsilon_{p}}^{c\left(T\right)}}{{\left\{ln\left[1-\frac{\sigma_{R}\left(T\right)}{\sigma_{p}}\right]-ln\left[W\left(T\right)-\frac{\sigma_{R}\left(T\right)}{\sigma_{p}}\right]\right\}}^{\frac{\text{1}}{m\left(T\right)}}} \end{array}$$ (32)

To determine the post-peak attenuation parameter λ, the convergence tolerance criterion [59] is used to identify the residual bearing stage and avoid interference from local data fluctuations. When the stress has dropped to 5% of the total post-peak strength loss, the coal is considered to have entered the residual bearing stage. The strain at this point is denoted as *εᵣ*, and the corresponding stress satisfies the 5% convergence tolerance condition:


$$\begin{array}{c} \sigma_{r}\left(T,\varepsilon \right)=0.05\left[\sigma_{p}\left(T\right)-\sigma_{R}\left(T\right)\right]+\sigma_{R}\left(T\right) \end{array}$$ (33)

By changing the constitutive equation of the post-peak section of the formula (15) in this paper, it can be obtained that:


$$\begin{array}{c} \sigma \left(T,\varepsilon \right)-\sigma_{R}\left(T\right)=\left[\sigma_{p}\left(T\right)-\sigma_{R}\left(T\right)\right]exp\left[-\lambda (\varepsilon -\varepsilon_{p}(T){)}^{\text{2}}\right] \end{array}$$ (34)

Therefore, when *ε* = *εᵣ*, we can get:


$$\begin{array}{c} 0.05\left[\sigma_{p}\left(T\right)-\sigma_{R}\left(T\right)\right]=\left[\sigma_{p}\left(T\right)-\sigma_{R}\left(T\right)\right]exp\left[-\lambda (\varepsilon -\varepsilon_{p}(T){)}^{\text{2}}\right] \end{array}$$ (35)

Taking the logarithm on both sides of Formula (35), we can get that λ is:


$$\begin{array}{c} \lambda =-\frac{ln20}{(\varepsilon_{r}-\varepsilon_{p}(T){)}^{\text{2}}} \end{array}$$ (36)

Finally, the solved parameters are substituted back to the original Equation (15), and the whole process piecewise damage constitutive model driven by real-time temperature established in this paper is:


$$\begin{array}{c} \sigma \left(T,\varepsilon \right)=\left\{ \begin{array}{c} @l@E_{\text{0}}\;exp\left(-\alpha \frac{T-T_{\text{0}}}{T_{max}}\right)\varepsilon^{c\left(T\right)}\left\{\frac{\sigma_{R}\text{(}T\text{)}}{\sigma_{p}}+\left(1-\frac{\sigma_{R}\text{(}T\text{)}}{\sigma_{p}}\right)exp\left[-{\left(\frac{F\left(T,\varepsilon \right)}{F_{\text{0}}\left(T\right)}\right)}^{m\left(T\right)}\right]\right\}(0\leq \varepsilon \leq \varepsilon_{p})\\ \sigma =\sigma_{R}\left(T\right)+\left(\sigma_{p}\left(T\right)-\sigma_{R}\left(T\right)\right)exp\left[-\lambda {\left(\varepsilon -\varepsilon_{p}\left(T\right)\right)}^{\text{2}}\right](\varepsilon_{p}\leq \varepsilon ) \end{array}\right. \end{array}$$ (37)

### 4.5. Model validation

**Fig 13:**
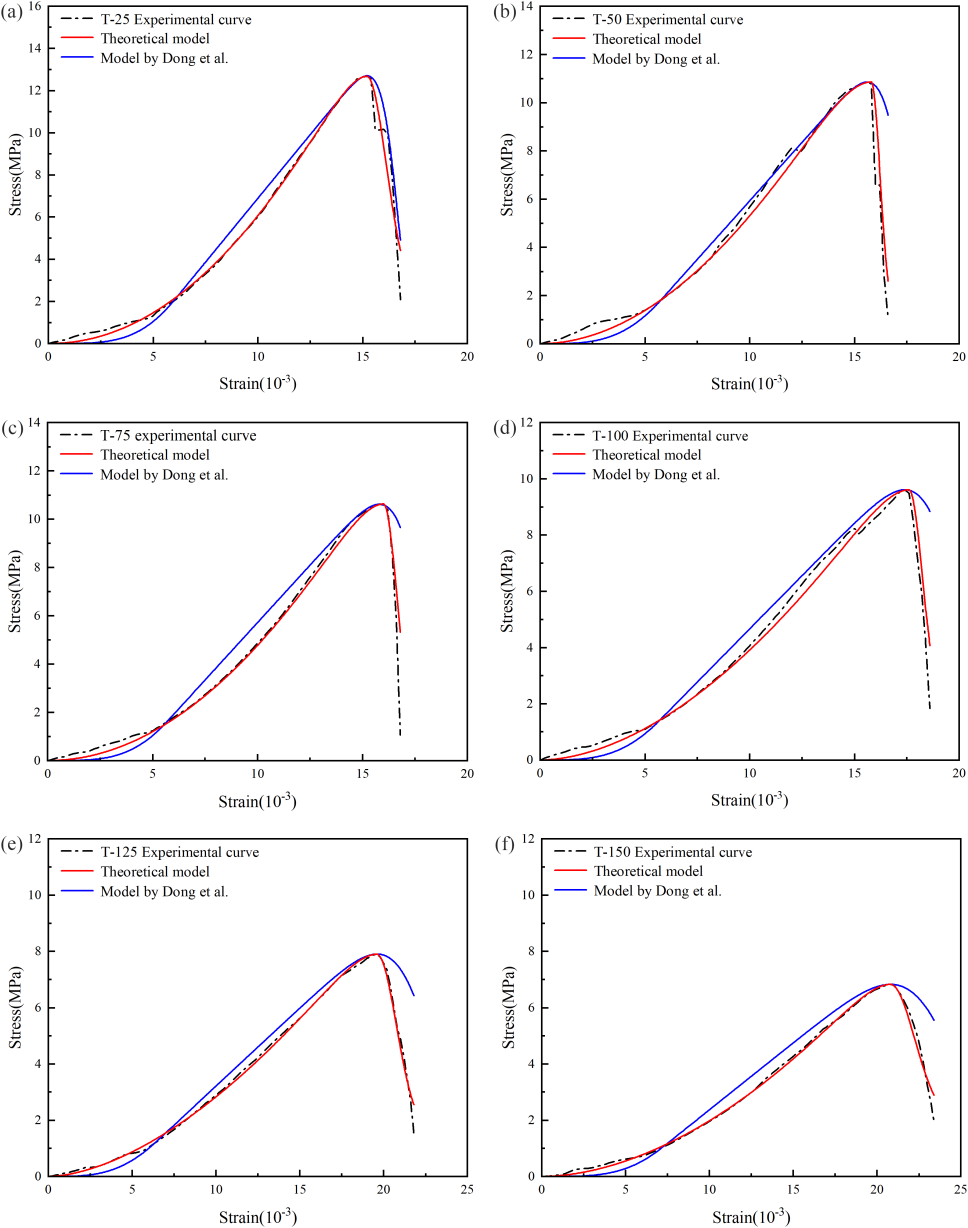
Theoretical curve comparison verification diagram. (a) 25 °C; (b) 50 °C; (c) 75 °C; (d) 100 °C; (e) 125 °C; (f) 150 °C.

Fig 13 compares the theoretical model curve with the experimental curve; all datasets used for curve verification are provided in S1 File. For comparison, the damage model of Dong et al. (2025) [27] is also included. The figure shows that the proposed model agrees well with the experimental data overall. Its advantage lies in accounting for the pore compaction effect of coal under temperature influence, which allows accurate description of the nonlinear compaction and linear elastic stages before the peak. In addition, the Gaussian-type decay function reconstructs the conventional Weibull model, enabling it to accurately capture the post-peak drop behavior.

The root mean square error (RMSE) and the coefficient of determination (R²) are used to evaluate the model [60]. RMSE measures the standard deviation between the calculated model and the experimental data, while R² indicates the degree of fit between the two. They are given as:


$$\begin{array}{c} {\text{R}}^{\text{2}}=1-\frac{\overset{n}{\underset{i=1}{\sum }}{\left({y_{i}}^{e}-{y_{i}}^{m}\right)}^{\text{2}}}{\overset{n}{\underset{i=1}{\sum }}{\left({y_{i}}^{\text{e}}-{\overset{―}{y}}^{e}\right)}^{\text{2}}} \end{array}$$ (38)

$$\begin{array}{c} \text{RMSE=}\sqrt{\frac{\text{1}}{n}\sum {\left(\overset{e}{\underset{i}{y}}-\overset{m}{\underset{i}{y}}\right)}^{\text{2}}} \end{array}$$ (39)

$\overset{e}{\underset{i}{y}}$ is the experimental data point, ${y_{i}}^{m}$ is the model prediction value, and ${\overset{―}{y}}^{e}$ is the mean value of the experimental data.

Table 4 lists the R² and RMSE values for both the proposed model and the model by Dong et al. (2025). For the proposed damage equation, the maximum RMSE is 0.5085 and the minimum R² is 0.9798. Compared with the proposed model, the maximum RMSE of Dong et al.’s model increased by 175.7%, while its minimum R² decreased by 135.9%.

**Table 4:** Comparison of fitting accuracy and error between two theoretical models.

*T*/°C	RMSE of equation	RMSE in Dong	R^2^ of equation	R^2^ in Dong
25	0.4324	0.6768	0.9893	0.9738
50	0.5085	1.4022	0.9798	0.8464
75	0.5038	1.1604	0.9796	0.8919
100	0.3741	1.0582	0.9855	0.8842
125	0.1360	0.8651	0.9972	0.8886
150	0.1395	0.6841	0.9962	0.9089

A closer look at the fitting accuracy in Table 4 shows that the R² values of the theoretical model range from 0.9796 to 0.9855 between 50 °C and 100 °C. This variation in accuracy confirms the physical authenticity of the thermomechanical coupling model presented here, and can be attributed to two main mechanisms. One is error transfer of basic parameters—the continuous evolution function *E*(*T*) for elastic modulus has some fitting deviation between 50 °C and 100 °C, and these fluctuations in the underlying parameters are passed on to the accuracy of the macroscopic theoretical model. The other is changes in microscopic mechanisms. Although the elastic modulus fits well at 75 °C, microscopic analysis in Section 3.3 shows that the inflection points for crack growth and macroscopic stress drop are 80.3 °C and 79.6 °C, respectively, placing 75 °C just before the onset of degradation. In this state, the macroscopic behavior of coal tends to be abrupt and locally unstable, while the theoretical model is a continuous, smooth function describing macroscopic damage—which inevitably leads to some deviation when capturing such nonlinear transitions.

In summary, the proposed model captures the coupled parameter transfer and nonlinear behavior of coal under thermal-mechanical loading. While minor fluctuations appear at certain temperatures, the overall average fit remains above 0.98, demonstrating its adaptability and accuracy.

## 5. Discussion

### 5.1. Macroscopic damage mechanism driven by real-time temperature

The findings show that under real-time temperature, the mechanical properties of coal degrade significantly, and the macroscopic failure mode shifts from axial splitting to a mixed tensile-shear pattern. PFC3D simulations confirm that high temperatures induce a large number of thermal damage cracks within the coal in advance. These pre-existing cracks merge with load-induced cracks during loading and cause the load-induced cracks to deflect. Such complex micro-scale friction and slip continuously consume external work, delaying the coalescence of the main crack. As a result, the strain interval between crack initiation and peak stress in the coal widens notably.

### 5.2. Accurate characterization of damage in the whole process of damage by the model

By incorporating initial pore compaction and the thermal sensitivity mechanism of the material, the pre-peak damage equation accurately captures the mechanical evolution of heated coal as reflected in the macroscopic stress-strain curve. For the post-peak behavior of coal under temperature effects, the conventional Weibull model decays too quickly after the peak, leading to considerable deviation. The damage constitutive model proposed here, based on a Gaussian attenuation function derived from the Gaussian kernel function, effectively reconstructs the post-peak attenuation path under temperature effects.

### 5.3. Engineering enlightenment to heat injection mining of CMB

The findings of this study show that real-time temperature not only promotes the thermal motion of gas molecules and their desorption from the coal, but the resulting thermal damage also induces a large number of tensile microcracks. These cracks provide natural seepage pathways for coalbed methane migration. However, the thermal damage caused by rising temperatures also significantly weakens the load-bearing capacity of the coal seam. Therefore, in practical applications of heat injection engineering, it is essential to consider how injection parameters dynamically affect reservoir mechanical stability and to develop a mining strategy that balances extraction efficiency with disaster prevention.

### 5.4. Limitations and future prospects

This study focuses on the real-time thermal-mechanical coupling effect under uniaxial compression, considering only a single temperature variable to reveal the baseline damage behavior of coal under unconfined conditions. However, coalbed methane reservoirs typically exist under complex stress conditions involving confining pressure and gas flow. Future work will introduce lateral confinement and gas seepage to further advance the theoretical framework of the damage constitutive model for coal under multi-field coupling.

## 6. Conclusion

(1) Within the temperature range studied, the mechanical properties of coal are negatively correlated with temperature. As temperature rises, both the elastic modulus and peak strength of coal progressively deteriorate. At 150 °C, the degree of deterioration reaches 69.45% for elastic modulus and 46.10% for peak strength.
(2) As temperature increases, both the macroscopic failure mode and the microscopic crack evolution of coal gradually shift from pure tensile failure to a mixed tensile-shear failure.
(3) The PFC3D numerical model was used to analyze, from a mesoscopic perspective, the fracture development characteristics of coal during the entire process from heating to failure under the influence of temperature. The results show that: (a) the cracks induced by thermal damage are mainly tensile cracks, and the proportion of tensile cracks increases with temperature; (b) the strain interval between the crack initiation point and the peak stress widens significantly as temperature rises, indicating a gradual increase in ductility.
(4) Based on the macroscopic mechanical properties of coal under real-time temperature conditions and the crack evolution observed in numerical simulations, a new segmented damage constitutive model was established. By introducing a compaction correction coefficient and constructing a Gaussian-type decay function, the traditional model was modified and reconstructed, significantly improving its ability to characterize the macroscopic mechanical behavior of coal.

## Supporting information

S1 File Raw data for mechanical tests, numerical simulations, and constitutive model verification. This single Excel file contains all original data generated in this study, which are organized into three worksheets. Sheet 1 includes the raw and processed macroscopic mechanical test data, such as stress-strain curves and failure parameters. Sheet 2 contains the numerical simulation datasets and model outputs, including simulation parameters and intermediate results. Sheet 3 presents the constitutive equation verification data. All data are original works of the authors and are fully available in this file.
(XLSX)

S2 File PFC simulation scripts. This file contains the customized FISH language scripts used for numerical simulations, including model setup, parameter assignment, and result output. All results, figures, and conclusions presented in this manuscript can be fully reproduced using only the provided S1 and S2 Supporting Information files.
(DOCX)
