# Supplementary material for: Damage characteristics and constitutive modeling of coal under real-time temperatures
Source: PLoS One. 2026 Jul 1;21(7):e0347468. doi: 10.1371/journal.pone.0347468 (PMC13322557; doi:10.1371/journal.pone.0347468)
Supplement: S2 File — This file contains the customized FISH language scripts used for numerical simulations, including model setup, parameter assignment, and result output. All results, figures, and conclusions presented in this manuscript can be fully reproduced using only the provided S1 and S2 Supporting Information files. (DOCX) [file pone.0347468.s002.docx]

new

domain extent -0.05 0.05 -0.05 0.05 -0.1 0.1 condition destroy

wall generate id 1 cylinder axis 0 0 1 base 0 0 -0.06 height 0.12 radius 0.025 cap false false onewall

wall generate id 2 plane dip 0 ddir 0 position 0 0 -0.05

wall generate id 3 plane dip 0 ddir 0 position 0 0 0.05

ball distribute porosity 0.25 box -0.025 0.025 -0.025 0.025 -0.05 0.05 ...

numbin 6 ...

bin 1 ...

radius 0.001 0.0015 ...

volumefraction 0.48 ...

group g1 ...

bin 2 ...

radius 0.001 0.0015 ...

volumefraction 0.08 ...

group g2 ...

bin 3 ...

radius 0.001 0.0015 ...

volumefraction 0.16 ...

group g3 ...

bin 4 ...

radius 0.001 0.0015 ...

volumefraction 0.12 ...

group g4 ...

bin 5 ...

radius 0.001 0.0015 ...

volumefraction 0.12 ...

group g5 ...

bin 6 ...

radius 0.001 0.0015 ...

volumefraction 0.04 ...

group g6

ball attribute density 2500 damp 0.7

def rezu

redao1=7.7

rezu1=1/(2*redao1*1e-3)

redao2=9.6

rezu2=1/(2*redao2*1e-3)

redao3=0.3

rezu3=1/(2*redao3*1e-3)

redao4=0.4

rezu4=1/(2*redao4*1e-3)

redao5=12.5

rezu5=1/(2*redao5*1e-3)

redao6=15.5

rezu6=1/(2*redao6*1e-3)

end

@rezu

configure thermal

cmat thermal default model thermalpipe property thres 65

contact groupbehavior and

cmat thermal add 1 model Thermalpipe property thres @rezu1 range group g1

cmat thermal add 2 model Thermalpipe property thres @rezu2 range group g2

cmat thermal add 3 model Thermalpipe property thres @rezu3 range group g3

cmat thermal add 4 model Thermalpipe property thres @rezu4 range group g4

cmat thermal add 5 model Thermalpipe property thres @rezu5 range group g5

cmat thermal add 6 model Thermalpipe property thres @rezu6 range group g6

cmat thermal default inherit thres off

ball thermal attribute sheat 0.720 range group g1

ball thermal attribute sheat 0.750 range group g2

ball thermal attribute sheat 0.880 range group g3

ball thermal attribute sheat 0.880 range group g4

ball thermal attribute sheat 0.880 range group g5

ball thermal attribute sheat 0.386 range group g6

cmat default model linear type ball-ball method deform emod 11e9 kratio 1.0 property fric 0.5

cmat default model linear type ball-facet method deform emod 22e9 kratio 1.0 property fric 0.5

set thermal off mechanical on

geometry set quyu

geometry generate cylinder axis 0 0 1 base 0 0 -0.06 height 0.12 radius 0.025 cap true true

ball delete range geometry quyu count odd not

cycle 1000 calm 10

set timestep scale

solve aratio 1e-3

set timestep auto

calm

wall delete walls range id 1

save model1

new

res model1

contact model linearpbond range contact type ball-ball

contact method bond gap 0.5e-4

contact method deform emod 2.65e9 krat 1.0

contact method pb_deform emod 2.65e9 krat 1.0

contact property pb_ten 9.3e6 pb_coh 46.5e6 pb_fa 0.0

contact property dp_nratio 0.5

contact property fric 0.5 range contact type ball-ball

ball attribute displacement multiply 0.0

contact property lin_force 0.0 0.0 0.0 lin_mode 1

ball attribute contactforce multiply 0.0 contactmoment multiply 0.0

cycle 1

solve aratio 1e-5

save model2

call 03.p3dat

new

res model2

;wall delete

call fracture.p3fis

@track_init

history id 1 @crack_num

ball attribute vel mul 0

ball attribute dis mul 0

ball attribute damp 0

ball thermal attribute thexp 10e-4 range group g1

ball thermal attribute thexp 10e-4 range group g2

ball thermal attribute thexp 14e-4 range group g3

ball thermal attribute thexp 14e-4 range group g4

ball thermal attribute thexp 18e-4 range group g5

ball thermal attribute thexp 18e-4 range group g6

ball thermal initialize temperature 298.15

ball thermal initialize temperature 298.15 range cylinder end1 0 0 -0.05 end2 0 0 0.05 radius 0.0 0.005

ball thermal fix range cylinder end1 0 0 -0.05 end2 0 0 0.05 radius 0.0 0.005

set mech on therm on

set timestep fix 4e-8

def wendubianhua;200计算时间步看做1min

loop n (1,10)

tem=13*n+298.15

command

ball thermal initialize temperature @tem range cylinder end1 0 0 -0.05 end2 0 0 0.05 radius 0.02 0.025

wall thermal initialize temperature @tem

ball thermal fix range cylinder end1 0 0 -0.05 end2 0 0 0.05 radius 0.02 0.025

cycle 100

endcommand

endloop

end

@wendubianhua

save model31

ball thermal free range cylinder end1 0 0 -0.05 end2 0 0 0.05 radius 0.0 0.005

cycle 500

save model32

; fname: fracture.p3fis

;

; Simple environment to track fragmentation in a BPM.

; Track LinearPBond model "bond_change" events and turn them into fractures.

; Use Fragment logic and Ball Result logic to record fragemnt ids

;

;==============================================================================

define add_crack(entries)

local contact = entries(1)

local mode = entries(2)

local frac_pos = contact.pos(contact)

local norm = contact.normal(contact)

local dfn_label = 'crack'

local frac_size

local bp1 = contact.end1(contact)

local bp2 = contact.end2(contact)

local ret = math.min(ball.radius(bp1),ball.radius(bp2));contact.method(contact,'pb_radius')

frac_size = ret

local arg = array.create(5)

arg(1) = 'disk'

arg(2) = frac_pos

arg(3) = frac_size

arg(4) = math.dip.from.normal(norm)/math.degrad

arg(5) = math.ddir.from.normal(norm)/math.degrad

if arg(5) < 0.0

arg(5) = 360.0+arg(5)

end_if

crack_num = crack_num + 1

if mode = 1 then

; failed in tension

crack_num_tension = crack_num_tension + 1

dfn_label = dfn_label + '_tension'

else if mode = 2 then

; failed in shear

crack_num_shear = crack_num_shear + 1

dfn_label = dfn_label + '_shear'

endif

global dfn = dfn.find(dfn_label)

if dfn = null then

dfn = dfn.add(0,dfn_label)

endif

local fnew = dfn.addfracture(dfn,arg)

dfn.fracture.prop(fnew,'age') = mech.age

dfn.fracture.extra(fnew,1) = bp1

dfn.fracture.extra(fnew,2) = bp2

crack_accum += 1

if crack_accum > 50

if frag_time < mech.age

frag_time = mech.age

crack_accum = 0

command

fragment compute

endcommand

; go through and update the fracture positions

loop for (local i = 0, i < 2, i = i + 1)

local name = 'crack_tension'

if i = 1

name = 'crack_shear'

endif

dfn = dfn.find(name)

if dfn # null

loop foreach local frac dfn.fracturelist(dfn)

local ball1 = dfn.fracture.extra(frac,1)

local ball2 = dfn.fracture.extra(frac,2)

if ball1 # null

if ball2 # null

local len = dfn.fracture.diameter(frac)/2.0

local pos = (ball.pos(ball1)+ball.pos(ball2))/2.0

if comp.x(pos)-len > xmin

if comp.x(pos)+len < xmax

if comp.y(pos)-len > ymin

if comp.y(pos)+len < ymax

if comp.z(pos)-len > zmin

if comp.z(pos)+len < zmax

dfn.fracture.pos(frac) = pos

end_if

end_if

endif

endif

endif

endif

endif

endif

endloop

endif

endloop

endif

endif

end

define track_init

command

dfn delete

ball result clear

fragment clear

fragment register ball-ball

endcommand

; activate fishcalls

command

set fish callback bond_break remove @add_crack

set fish callback bond_break @add_crack

endcommand

; reset global variables

global crack_accum = 0

global crack_num = 0

global crack_num_tension = 0

global crack_num_shear = 0

global track_time0 = mech.age

global frag_time = mech.age

global xmin = domain.min.x()

global ymin = domain.min.y()

global xmax = domain.max.x()

global ymax = domain.max.y()

global zmin = domain.min.z()

global zmin = domain.min.z()

end

;==============================================================================

; eof: fracture.p3fis

res model32

def wall_point

wp3 = wall.find(3) ;shang

wp2 = wall.find(2) ;xia

end

@wall_point

[wlz=wall.pos.z(wp3)-wall.pos.z(wp2)]

def jiance

whilestepping

force1 = -wall.force.contact.z(wp3)

force2 = wall.force.contact.z(wp2)

wszz = -0.5*(force1+force2)/(math.pi*0.025*0.025)

wezz = -(wall.pos.z(wp3)-wall.pos.z(wp2)-wlz)/wlz

end

define loadhalt_wall

loadhalt_wall = 0

local abs_stress = math.abs(wszz)

global peak_stress = math.max(abs_stress,peak_stress)

if abs_stress < peak_stress*peak_fraction

loadhalt_wall = 1

end_if

end

;cyc 10000

wall attribute zvelocity -1 range id 3

wall attribute zvelocity 1 range id 2

history id 2 @wszz

history id 3 @wezz

set thermal off mechanical on

set timestep auto

SET @peak_fraction = 0.6

solve fishhalt @loadhalt_wall

list @peak_stress

save model4
